# Methylprednisolone-Induced Symptomatic Sinus Bradycardia in a Multiple Sclerosis Patient: A Case Report

**DOI:** 10.7759/cureus.21443

**Published:** 2022-01-20

**Authors:** Mohammed A Miqdad, Abdullah Mohamad, Fawaz Ali, Abdul Rahman Mourad, Abdullah Alamri

**Affiliations:** 1 Internal Medicine, Dr. Sulaiman Al-Habib Hospital, Khobar, SAU; 2 Internal Medicine, Dr. Sulaiman Al-Habib Medical Group, Khobar, SAU; 3 Cardiology, Dr. Sulaiman Al-Habib Hospital, Khobar, SAU; 4 Neurology and Neurocritical Care, King Fahd Hospital of the University, Imam Abdulrahman Bin Faisal University, Khobar, SAU

**Keywords:** pulse therapy, corticosteroid, multiple sclerosis flare-up, multiple sclerosis, arrhythmia, bradycardia, methylprednisolone

## Abstract

Intermittent high-dose methylprednisolone therapy is widely used for various autoimmune conditions treatment. Common side effects are well known and monitored carefully during therapy. Although cardiovascular adverse events are uncommon, they have been increasingly reported in the literature. This is a case of a 30-year-old female who developed symptomatic sinus bradycardia after receiving three grams of intravenous methylprednisolone pulse therapy for multiple sclerosis flare-ups. Her pulse rate reached 40bpm, together with lightheadedness and chest tightness. An electrocardiogram confirmed sinus bradycardia, for which she was initially managed by splitting the methylprednisolone dose in half; however, 12 hours later, the heart rate decreased further to 35bpm, and her symptoms worsened.

Subsequently, the medicine was omitted, and the patient shifted to the intensive care unit for close observation and monitoring. She was treated conservatively with close observation resulted in a gradual normalization of the heart rate. The diagnosis of methylprednisolone pulse-induced bradycardia was made after excluding other common etiologies of sinus bradycardia. This case report aims for careful cardiovascular monitoring in patients receiving high doses of methylprednisolone due to the dose-dependent cardiovascular risks.

## Introduction

Multiple sclerosis (MS) exacerbation is defined as a patient-reported or objectively observed event typical of an acute inflammatory demyelinating event in the central nervous system, current or historical, with a duration of at least 24 hours in the absence of fever or infection, based on the revised McDonald criteria [1]. High-dose intravenous (IV) methylprednisolone therapy (1000mg) has been the standard line of management in multiple sclerosis acute exacerbations for three to seven days [1]. However, there is a risk of cardiovascular adverse events associated with glucocorticoid use increase with higher doses and intravenous administration, reaching up to 82% of patients receiving high-dose corticosteroids [2]. 

Additionally, oral pulse steroids have been reported to cause bradycardia and other arrhythmias [2]. Glucocorticoid use was reported to be associated with certain cardiovascular risks, including myocardial infarction, stroke, heart failure, and a two-fold risk increase in atrial fibrillation or flutters based on a population-based case-control study [3]. Sinus bradycardia after high-dose methylprednisolone is an uncommonly reported side effect [2]. Nonetheless, it was first reported in five patients in 1986 who received high dose methylprednisolone for rheumatoid arthritis [4]. 

## Case presentation

**Figure 1:**
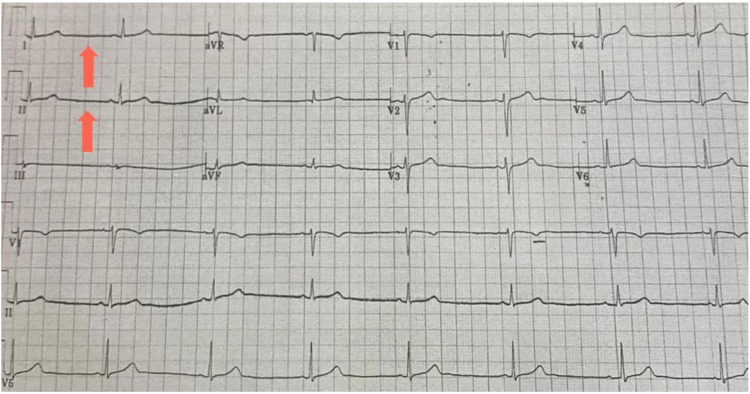
An ECG at rest showed sinus bradycardia with no other changes

This case report is about a 30-year-old lady with a two-year history of relapsing-remitting multiple sclerosis involving the brain and spinal cord. Her expanded disability status scale (EDSS) is 1 in the form of recurrent numbness. She presented to the emergency department due to numbness, blurry vision, and dizzy spells for the last five days, for which she was admitted for intravenous methylprednisolone pulse therapy in order to treat a new relapse complicating a new tumofactive lesion. After obtaining a brain MRI which confirmed MS active lesions, she was started on methylprednisolone sodium succinate 1 g diluted in 250 mL of 0.9% normal saline intravenously over four hours. On the third day, she developed asymptomatic sinus bradycardia documented by electrocardiogram (Figure 1) with normal blood pressure, O2 saturation, and respiratory rate. Notably, she had no prior cardiac disease, and cardiovascular and pulmonary systems were unremarkable.

**Figure 2:**
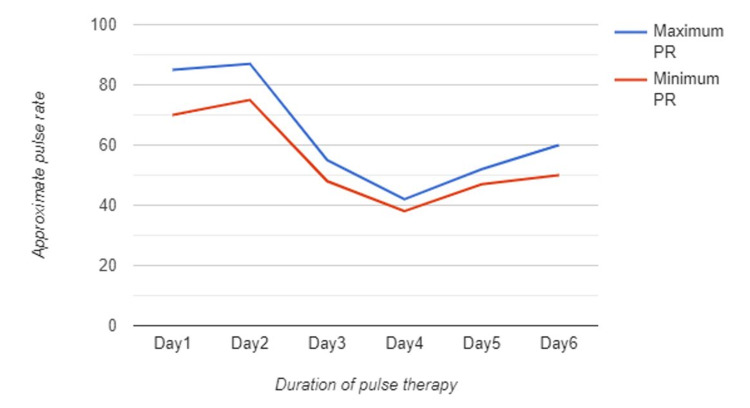
Maximum and minimum pulse rate recording before and after pulse therapy initiation Gradually decreased pulse rate during the administration of IV Methylprednisolone, starting on day 2, and slowly normalized after omitting the pulse therapy on day 5 to 6

Subsequently, the methylprednisolone dose was split to 500mg twice per day, and all blood work-related bradycardia was ordered, along with continuous ECG monitoring and echocardiogram. Upon the fourth day of pulse therapy, the heart rate dropped further to 35-38bpm, for which she started to experience dizziness, malaise, and chest tightness. Nonetheless, other vital signs were recorded within normal; BP was 127/82, O2 saturation was 98%, and RR was 15 breaths/minutes. As a result, methylprednisolone was omitted, and the patient was shifted to the intensive care unit for close observation and cardiac monitoring. Consequently, her symptoms improved, and her pulse rate gradually normalized with no pharmacological intervention. Figure 2 exhibits a gradually decreased pulse rate during the administration of pulse therapy, starting on day 1, and a progressively normalized pulse rate after omitting the pulse therapy on Day 5 to 6. 

On the other hand, her echocardiography, together with laboratory investigations, was normal, apart from a slightly low TSH level, which is expected after high-dose steroid therapy (Table 1). The patient was discharged with regular follow-up and instructed to seek medical advice immediately if symptoms return. Upon follow-up, the patient was asymptomatic, in a good clinical condition, and an ECG showed normal sinus rhythm. The diagnosis of methylprednisolone-induced bradycardia was made after excluding other common causes of sinus bradycardia. The vasovagal response was initially proposed but it would typically occur within the first day of infusion, in addition to normal blood pressure recording during admission. We encourage physicians to carefully observe patients undergoing steroid pulse therapy, mainly if high doses are demanded. 

**Table 1:** Laboratory Investigations WBC: White blood cells; TSH; Thyroid-stimulating hormone; AST; Aspartate aminotransferase; ALT: Alanine aminotransferase; T. bilirubin: Total bilirubin

Parameter	Result	Reference range	Parameter	Result	Reference range
WBC	6.1	4-11 × 10^9^/l	TSH	0.231	0.5-5 mlU/l
Hemoglobin	11.2	11-13 (g/dl)	AST	23	10-45 u/l
Serum Na	137	135-145 mmol/l	ALT	50	10-55 u/l
Serum K	4.2	3.5-5.1 mmol/l	T. bilirubin	11	5-18 µmol/l
Corrected Ca	2.3	2.1-2.4 mmol/l	Creatinine	78	50-110 µmol/l
Serum Mg	0.85	0.7-1.1 mmol/l	Urea	4.2	3.5-5.5 mmol/l
Serum PO4^-3^	0.91	0.8-1.2 mmol/l	D-dimer	0.36	<0.5 mg/l
Blood glucose	4.1	3.5-5.1 mmol/l	Urine analysis	Normal	

## Discussion

The mechanism of steroid-induced bradycardia remains unclear, but certain mechanisms have been thought to be related. As reported in animal studies, the high-dose methylprednisolone can directly affect the cardiac myocyte by altering cardiovascular sensitivity to catecholamines. In humans, corticosteroids can influence sudden electrolyte shifts, leading to cardiac arrhythmias, such as bradycardia. On the other hand, it is hypothesized that pulse corticosteroids might induce sodium and water physiologic changes resulting in plasma volume expansion and activating low-pressure baroreceptors [2].

Furthermore, various factors are linked to bradycardia induced by corticosteroid pulse therapy, such as rapid rates of IV corticosteroid infusion (particularly under 30 minutes) and renal or cardiac comorbidities. In patients without comorbidities, corticosteroids are unlikely to cause bradycardia and demonstrate good tolerability. Sinus bradycardia was also reported on the fifth day after oral pulse corticosteroid therapy initiation, and onset usually can vary from one to seven days [2].

Additionally, high-dose oral pulse corticosteroids of 1,250 mg daily for five days were associated with symptomatic bradycardia in a case involving a 45-year-old lady. She was diagnosed with MS flare-up and started to experience chest pain with mild dyspnea three days after completing the course. Likewise, she improved with close monitoring and a conservative approach [5]. Besides, high-dose intravenous methylprednisolone was also reported to induce myocardial infarction, hypotension, and asystole [6-7].

## Conclusions

Cardiovascular outcomes secondary to methylprednisolone, particularly bradycardia, have been increasingly reported, although it is a rare consequence. Nevertheless, bradycardia is commonly sinus, asymptomatic, and followed by spontaneous recovery. Indeed, most physicians would prescribe corticosteroids at some point, either orally or by an intravenous route. Therefore, the heart rate variation should be monitored closely for patient safety. More studies are needed to elaborate on the pathophysiology in such patients.
